# Behavioral and cellular responses to circadian disruption and prenatal immune activation in mice

**DOI:** 10.1038/s41598-023-34363-w

**Published:** 2023-05-13

**Authors:** Tara C. Delorme, William Ozell-Landry, Nicolas Cermakian, Lalit K. Srivastava

**Affiliations:** 1 Douglas Mental Health University Institute, 6875 Boulevard LaSalle, Montréal, QC H4H 1R3 Canada; 2 Integrated Program in Neuroscience, McGill University, Montréal, QC H3A 2B4 Canada; 3 Department of Psychiatry, McGill University, Montréal, QC H3A 1A1 Canada

**Keywords:** Immunochemistry, Risk factors, Circadian rhythms and sleep, Autism spectrum disorders, Schizophrenia

## Abstract

Most individuals with neurodevelopmental disorders (NDDs), including schizophrenia and autism spectrum disorders, experience disruptions in sleep and circadian rhythms. Epidemiological studies indicate that exposure to prenatal infection increases the risk of developing NDDs. We studied how environmental circadian disruption contributes to NDDs using maternal immune activation (MIA) in mice, which models prenatal infection. Pregnant dams were injected with viral mimetic poly IC (or saline) at E9.5. Adult poly IC- and saline-exposed offspring were subjected to 4 weeks of each of the following: standard lighting (LD1), constant light (LL) and standard lighting again (LD2). Behavioral tests were conducted in the last 12 days of each condition. Poly IC exposure led to significant behavioral differences, including reduced sociability (males only) and deficits in prepulse inhibition. Interestingly, poly IC exposure led to reduced sociability specifically when males were tested after LL exposure. Mice were exposed again to either LD or LL for 4 weeks and microglia were characterized. Notably, poly IC exposure led to increased microglial morphology index and density in dentate gyrus, an effect attenuated by LL exposure. Our findings highlight interactions between circadian disruption and prenatal infection, which has implications in informing the development of circadian-based therapies for individuals with NDDs.

## Introduction

Neurodevelopmental disorders (NDDs), such as schizophrenia (SCZ) and autism spectrum disorders (ASD), are multifactorial in nature, whereby multiple interacting risk factors are required to trigger disease onset and contribute to symptomatology^1,2^. These risk factors are thought to pathologically disrupt normal brain development^3^. Studying how multiple risk factors for NDDs interact with each other, rather than studying single risk factors, is necessary and becoming a recurrent theme in research^4^. One such risk factor is prenatal infection, which has been strongly associated with an offspring’s risk of developing NDDs^5,6^. Exposure to prenatal infection in the first trimester of pregnancy has been associated with an elevated risk of offspring to develop SCZ^7^ and ASD^8^.

Prenatal infection is believed to act as a primer for disease, and in combination with other risk factors may result in the full display of symptoms^9^. Prenatal infection can be modelled in animals using a maternal immune activation (MIA) protocol^10^, where an infection is simulated in pregnant dams using viral mimetic polyinosinic:polycytidylic acid (poly IC). MIA results in behavioral deficits related to NDDs^11^ (e.g., reduced sociability^12^), and cellular dysfunction (e.g., altered microglia properties^13^). Microglia are immune surveillant cells that colonize the fetal brain^14^ and remain in the central nervous system until adulthood. Microglia contribute to sculpting neural circuits by pruning synapses and remodeling circuits during normal brain development and disease^15,16^. Given that the alterations in microglia following MIA persist until adulthood^13^, they are expected to play key roles in NDDs^17^.

A less commonly discussed perturbation in the context of NDDs is the disruption of circadian rhythms. Circadian rhythms are daily (~ 24 h) cycles in behavior (e.g., sleep, mood) and physiology (e.g., certain hormones and genes). These rhythms are generated through clock mechanisms present in most mammalian cells^18^. To maintain synchrony with our environment, these endogenously generated rhythms need to be ‘reset’ daily by cyclic cues. The strongest rhythmic environmental cue is light exposure^19^. Disruption to the circadian timing system, most commonly through the inappropriate exposure to light, can be detrimental to our mental and physical health, especially if experienced chronically^20,21^. For example, most shift workers regularly experience circadian disruption^22^, and are more at risk for cardiovascular disease^23^, cancer^24^, sleep disorders^25^ and negative mental health outcomes^26^.

Not only do about 80% of individuals with SCZ and ASD exhibit various disruptions in sleep, rest/activity rhythms, daily hormone rhythms and circadian clock gene expression^27–29^, but sleep is also inversely correlated with the severity of psychosis^30^. Circadian disturbances are similarly reported in animal models based on genetic risk factors for SCZ^31^ and ASD^32^, and using an MIA protocol in mice^33^. In many individuals with NDDs, circadian disturbances precede the onset of psychosis^30,34^, which supports a role for circadian disruption as a risk factor for NDDs. This hypothesis is reinforced in a genetic mouse model for SCZ, where SCZ-related behaviors worsened after altered light exposure ^35,36^. If circadian disruption is a risk factor for NDDs, individuals with NDDs may be vulnerable to the effects of environmental circadian disruption (e.g., shift work), in ways that affects the expression of symptoms experienced. This is especially problematic given that approximately 28% of the Canadian labor force and 40% of the American labor force work mostly during non-standard times^37,38^.

In this study, we aimed to explore if circadian disruption caused by inappropriate light exposure is a risk factor for NDDs and if MIA (first risk factor in-utero) combined with exposure to circadian disruption (second risk factor in adulthood) would synergistically induce behavioral and microglial deficits in offspring. To explore this, we used an MIA protocol in mice and characterized behavior before and after the mice were exposed to constant light (LL) in adulthood and then characterized microglia after re-exposure to LL or standard lighting. Sex differences are observed in the prevalence, age of onset, and severity and profile of symptoms of individuals with SCZ and ASD^39,40^. Specifically, SCZ is approximately 1.4 times more prevalent in males^41^, and ASD is 3–4 times more likely to be diagnosed in males than females^42,43^. Sex differences are also apparent in rodent models of MIA, where males tend to exhibit stronger behavioral and cellular phenotypes than females^44^ and MIA has been shown to lead to sex-specific changes in microglial gene expression^45^. For these reasons, we have incorporated female mice in our experiments when feasible.

## Materials and methods

For detailed materials and methods, see the Supplementary Information.

### Animals

Animal use was in accordance with the guidelines of the Canadian Council of Animal Care and was approved by the McGill University Animal Care Committee. Authors complied with the ARRIVE guidelines. Further details are in Supplementary Methods.

### Maternal immune activation (MIA) protocol

MIA was performed as previously described^33^. On embryonic day 9.5 (E9.5), pregnant dams were intraperitoneally injected with poly IC dissolved in double-distilled water based on body weight (5 mg/kg; lot 1: 086M4045V; Sigma-Aldrich, St. Louis, MO, USA) or sterile saline solution. Experiments were replicated using a second lot of poly IC (lot 2: 096M4023V). We have previously shown that these lots of poly IC induced an immune response in pregnant dams^33^. Litters of poly IC- and saline-exposed dams did not differ in number of pups (Supplementary Fig. 1A,B). Further details are in Supplementary Methods.

### Experimental timeline

**Figure 1:**
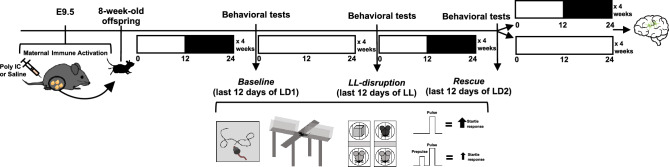
Experimental timeline. On E9.5 pregnant dams were intraperitoneally injected with poly IC or saline. Poly IC- and saline-exposed offspring were aged to adulthood and were successively subjected to 4 weeks of each of the following conditions: standard lighting (LD1), constant light (LL) and standard lighting again (LD2). Behavioral tests were conducted in the last 12 days of each condition. Mice underwent the open field test, the elevated plus maze, the three-chamber social interaction test and prepulse inhibition of acoustic startle. After the last behavioral test, poly IC- and saline-exposed males were placed back into standard lighting (LD) or LL for 4 weeks before their brains were harvested for immunohistochemistry.

The experimental protocol is depicted in Fig. 1**.** Baseline behavior was assessed by subjecting mice to behavioral testing in the last 12 days of a 4-week exposure to standard lighting (LD1). Mice were tested once again, but this time in the last 12 days of a 4-week exposure to LL, a lighting condition known to disrupt circadian rhythms. Finally, to ‘rescue’ LL-induced deficits, we tested mice a third time in the last 12 days of a 4-week exposure to standard lighting again (LD2). After the conclusion of behavioral testing, male mice were placed back into either regular lighting (LD) or LL for 4 more weeks before their brains were harvested for immunohistochemistry. At this stage, due to space constraints, female poly IC- and saline-exposed mice were euthanized and not used for subsequent experiments. Further details are in Supplementary Methods.

### Behavioral outcomes

Detailed information for each behavioral test is in Supplementary Methods.

#### Open field test

The open field test was used to assess spontaneous locomotor activity and anxiety-like behavior^46^. Measures such as horizontal activity (number of horizontal beam breaks), total distance traveled and thigmotaxis (time spent in the outer edges of the apparatus divided by time spent in the center) were analyzed^47^.

#### Elevated plus maze (EPM) test

Anxiety-like behavior was assessed using the elevated plus maze (EPM)^48^. The apparatus consisted of a plus-shaped maze. Two opposing arms (closed arms) were enclosed by 10 cm high walls, while the other two opposing arms (open arms) did not have walls. See EPM formula below.

$${\text{EPM}}\,{\text{formula = }}\frac{{{\text{Time}}\,{\text{spent}}\,{\text{in}}\,{\text{open}}\,{\text{arms (s)}}}}{{{\text{Time}}\,{\text{spent}}\,{\text{in}}\,{\text{open}}\,{\text{arms (s) + Time}}\,{\text{spent}}\,{\text{in}}\,{\text{closed}}\,{\text{arms (s)}}}} \times {100}{\text{.}}$$

#### Three-chamber social interaction test

Social preference and social memory were assessed using the three chamber social interaction test^49^. In the social preference phase, a mouse that our experimental mouse had never interacted with before, called stranger 1, was placed under one of the wire containers, and an object under the other wire container. See social preference formula below.

$$\text{Social preference formula = }\frac{\text{Time spent in Stranger 1 zone}}{\text{Time spent in Stranger 1 zone+Time spent in Object zone}}.$$

In the social memory phase, the object was replaced by a novel mouse, again being a mouse that the experimental mouse had never interacted with before, called stranger 2. See social memory formula below.

$$\text{Social memory formula}\text{ = }\frac{\text{Time spent in Stranger 2 zone}}{\text{Time spent in Stranger 2 zone+Time spent in Stranger 1 zone}}.$$

#### Prepulse inhibition of acoustic startle (PPI)

PPI is a measure of sensory-motor gating^50^. As described previously, mice were placed into a cylindrical Plexiglass enclosure, mounted on a Plexiglass base. Each session consisted of 50 trials, some of which only had a 120-dB startle noise burst, with others had a prepulse presented before the startle noise burst. A piezoelectric accelerometer fixed to the Plexiglass base was used to detect and transduce motion resulting from the animal’s startle response. Percent PPI was calculated as follows.

$${\text{\% prepulse }}\,{\text{inhibition = }}100 - \left( {{ }\frac{{{\text{Startle}}\,{\text{amplitude}}\,{\text{on}}\,{\text{prepulse}}\,{\text{trials}}}}{{{\text{Startle}}\,{\text{amplitude}}\,{\text{on}}\,{\text{pulse}}\,{\text{alone}}\,{\text{trials}}}}} \right) \times { 100}{\text{.}}$$

### Microglia characterization

After the last behavioral test, poly IC- and saline-exposed male offspring were placed into LD or LL for 4 weeks before their brains were harvested for immunohistochemistry. Further details are in Supplementary Methods.

#### Microglia visualization and analysis

Microglia from the dorsal hippocampus (both the dentate gyrus and CA1) and medial PFC were imaged using Z-stacks at a 20× magnification. Each stack contained ~ 30 slices (1 μm each). The images were analyzed using Fiji ImageJ software, while being blinded to the experimental conditions. Microglia density and morphology were characterized essentially as described previously^13^. A more reactive microglial profile would include a higher morphological index, a less circular cell body, greater density, and reduced spacing index. Further details are in Supplementary Methods.

### Statistics

Data were analyzed and graphed using Prism version 9 (GraphPad). Differences were considered significant if *p* < 0.05. Details on the statistical analyses are available in the Supplementary Methods.

## Results

### Comparison and validation of poly IC lots

The behavioral data were collected from two different cohorts of mice, each treated with a different lot of poly IC. The results from the first cohort (poly IC lot 1) are presented in the main manuscript, and the results from the second cohort (poly IC lot 2) are presented in the Supplementary Information. We previously confirmed that both lots of poly IC used in the study induced significant inflammatory responses in pregnant dams^33^, although cytokine and chemokine levels induced by poly IC lot 1 were ~ 2 times higher compared to poly IC lot 2. Additionally, using poly IC lot 1 but not lot 2, there was a significant treatment x age interaction, where poly IC exposure led to decreased weight in adulthood for both males and females (males: F_(1, 84)_ = 7.946, *p* = 0.0060; post hoc with adulthood poly IC versus saline, *p* = 0.0122; females: F_(1, 28)_ = 7.385, *p* = 0.0112; post hoc with adulthood poly IC versus saline, *p* = 0.0440) (Supplementary Fig. 1C–F).

### Behavior

#### Hyperactive phenotype following prenatal poly IC exposure in males.

**Figure 2:**
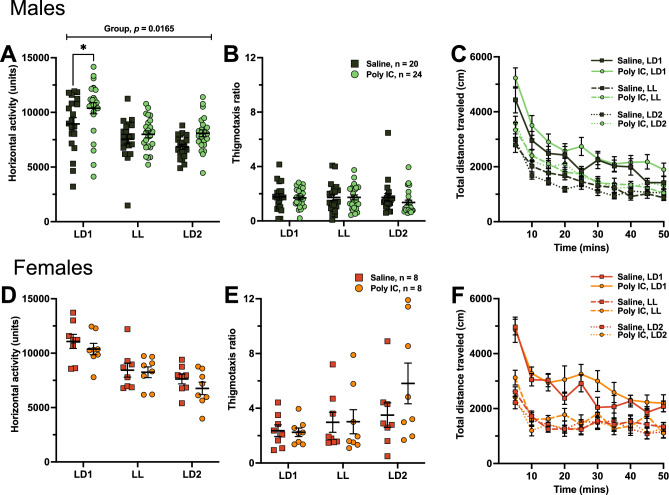
Hyperactive phenotype in poly IC-exposed males. Spontaneous locomotor activity was measured in the open field test. Horizontal activity (**A**,**D**), thigmotaxis (**B**,**E**) and total distance traveled (**C**,**F**) were assessed in males (**A–C**) and females (**D–F**). For panels (**A**,**B**,**D**,**E**) data points represent individual mice and are presented as mean ± SEM. Two-way ANOVAs (factors treatment x lighting with Tukey’s post-hoc comparisons) were conducted. For panels (**C**) and (**F**), group averages ± SEM are shown over each 10-min bin of the test. Three-way ANOVAs (factors treatment × lighting × time) were conducted. See Supplementary Table 1 and 2 for full statistics. *p < 0.05 (post hoc).

Spontaneous locomotion was assessed using the open field test. Using poly IC lot 1, poly IC-exposed males showed a hyperactive phenotype compared to saline-exposed males. This was seen in an increased amount of horizontal activity (main effect of treatment, F_(1, 42)_ = 6.233, *p* = 0.0165; post hoc saline versus poly IC under LD1, *p* = 0.0456) (Fig. 2A) and increased total distance traveled (main effect of treatment, F_(1, 42)_ = 7.398, *p* = 0.0095) (Fig. 2C). The complete statistical parameters from the behavioral studies are listed in Supplementary Tables 1–4. No significant treatment x lighting interactions were observed, and no significant differences were seen in thigmotaxis ratio (Fig. 2B). Interestingly, no significant differences were observed in locomotion using poly IC lot 2 in males (Supplementary Fig. 2A–C), and no differences were observed in females using either lot (Fig. 2D–F, Supplementary Fig. 2D–F). Taken together, no significant interactions were observed in spontaneous locomotion, but poly IC exposure induced a hyperlocomotive phenotype in males while using poly IC lot 1.

#### Limited effects of prenatal poly IC exposure on anxiety-like behavior

**Figure 3:**
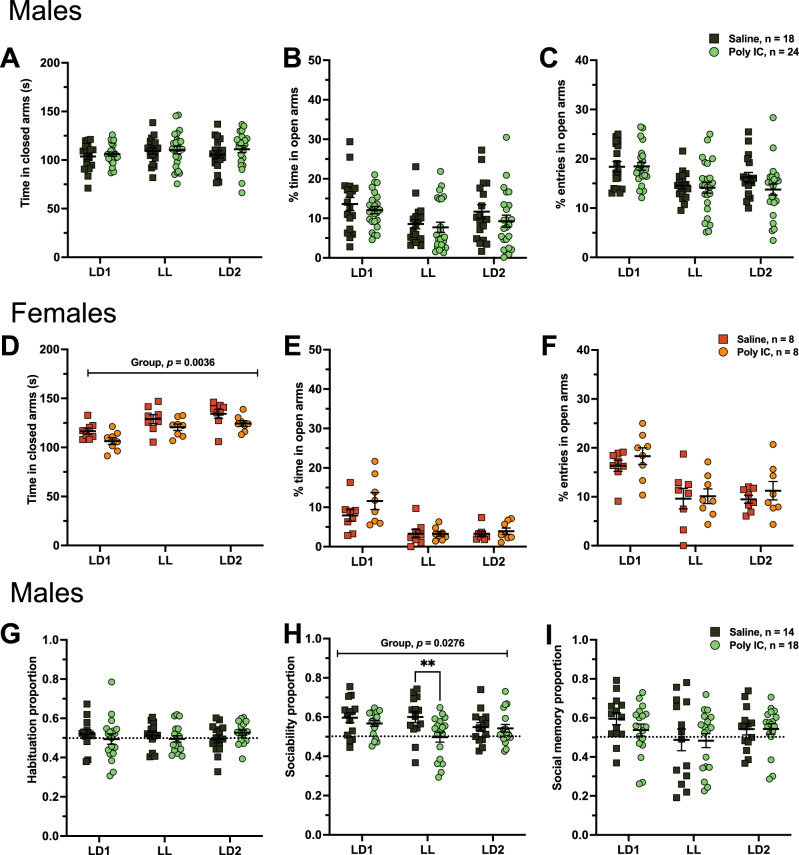
Prenatal poly IC led to limited effects in EPM and reduced sociability after LL. The elevated plus maze was used to assess anxiety-like behavior. Time in closed arms (**A**,**D**), percent time in open arms (**B**,**E**) and percent entries in open arms (**C**,**F**) were assessed in males (**A**–**C**) and females (**D**–**F**). The three-chamber social interaction test was used to assess sociability and social memory. Preference proportions were assessed for the habituation phase (**G**), sociability phase (**H**) and social memory phase (**I**) in males. Data points represent individual mice, and are presented as mean ± SEM. Two-way ANOVAs (factors treatment × lighting with Tukey’s post-hoc comparisons) were conducted. See Supplementary Table 1 and 2 for full statistics. **p < 0.01 (post hoc).

Anxiety-like behavior was measured using the EPM test. In males, no significant differences were observed in the time spent in the closed arms, and percent time spent and percent entries in open arms with either lot of poly IC (Fig. 3A–C, Supplementary Fig. 3A–C). Thus, neither poly IC exposure, LL exposure, or their interaction had a significant effect in anxiety-like behavior in males. This is consistent with the lack of differences in thigmotaxis in the open field test (Fig. 2B, Supplementary Fig. 2B).

In females, although poly IC-exposed mice showed no significant treatment x lighting interactions in the tested parameters, they exhibited a significant decrease in time spent in closed arms using lot 1 but not lot 2 (main effect of treatment; F_(1, 13)_ = 12.47, *p* = 0.0036) (Fig. 3D, Supplementary Fig. 3D). However, there was no difference in percent time spent and percent entries in open arms while using either lot (Fig. 3E,F, Supplementary Fig. 3E,F)*.* Thus, some evidence supports that poly IC exposure in females led to less anxiety-like behavior, but this is not supported by percent time spent or percent entries in open arms, nor thigmotaxis, and was not replicated using poly IC lot 2.

#### Reduced sociability after LL exposure in poly IC-exposed males

Social interaction was measured using the three-chamber social interaction test. Due to practical reasons and time constraints, only a subset of males (and no females) was used in this test compared to the other behavioral tests. For the habituation phase, there were no significant differences in the amount of time spent in each chamber using either of the poly IC lots (Fig. 3G, Supplementary Fig. 3G). During the sociability phase, poly IC exposure led to reduced sociability in poly IC lot 1 (main effect of treatment, F_(1, 90)_ = 6.172, *p* = 0.0276) but not lot 2 (Fig. 3H, Supplementary Fig. 3H). The effect reported in lot 1 was expected and has been reported for prenatal poly IC exposure^51^. Interestingly, post hoc analyses revealed that the overall reduction in sociability caused by poly IC was driven by a decreased sociability exhibited after LL exposure (*p* = 0.0081) (Fig. 3H), and after conducting post hoc comparisons on the lot 2 data, we saw a similar trend when we compared poly IC versus saline under LL (*p* = 0.0985) (Supplementary Fig. 3H). During the social memory phase, there were no significant differences in social memory (Fig. 3I, Supplementary Fig. 3I). In sum, LL exposure acted to uncover a reduced sociability phenotype in the MIA mice.

#### Deficits in PPI following prenatal poly IC exposure

**Figure 4:**
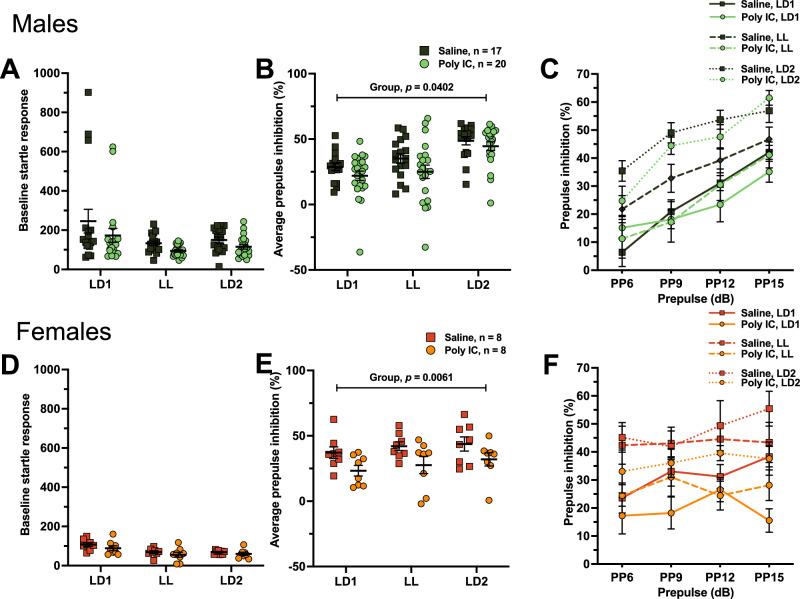
Deficits in PPI following prenatal poly IC exposure. Prepulse inhibition of acoustic startle (PPI) was used to assess sensory motor gating. Baseline startle response (**A**,**D**), average PPI (%) (**B**,**E**) and PPI (%) across each prepulse levels (**C**,**F**) were assessed in males (**A**–**C**) and females (**D**–**F**). For panels (**A**,**B**,**D**,**E**) data points represent individual mice and are presented as mean ± SEM. Two-way ANOVAs (factors treatment x lighting with Tukey’s post-hoc comparisons) were conducted. For panels (**C**) and (**F**)**,** group averages ± SEM are shown over prepulse level. Three-way ANOVAs (factors treatment × lighting × time) were conducted. See Supplementary Tables 1 and 2 for full statistics.

Sensory-motor gating was measured using the prepulse inhibition of acoustic startle (PPI) test. The baseline startle response was not significantly different between groups for either lots (Fig. 4A, Supplementary Fig. 4A). We found a significant three-way interaction for both lots of poly IC (prepulse × lighting × treatment: lot 1: F_(1, 40)_ = 3.637, *p* = 0.008; lot 2: F_(1, 40)_ = 4.823, *p* = 0.047) (Fig. 4C, Supplementary Fig. 4C) and we decomposed this three-way interaction by performing two-way ANOVAs between lighting x treatment. Consistent with the literature on SCZ^52^ and ASD^53^, poly IC exposure induced a decrease in percent PPI (main effect of treatment: lot 1: F_(1, 40)_ = 4.498, *p* = 0.04; lot 2: F_(1, 40)_ = 3.827, *p* = 0.02), without significant treatment x lighting interactions (Fig. 4B, Supplementary Fig. 4B). Thus, poly IC exposure led to a reduction in PPI, indicative of deficits in sensory-motor gating. The lack of treatment x lighting interactions or main effects of lighting suggests that the addition of LL exposure did not influence sensory-motor gating in males.

In females, no differences in baseline startle response were reported (Fig. 4D, Supplementary Fig. 4D). The three-way ANOVAs (prepulse × lighting × treatment) were not significant (Fig. 4F, Supplementary Fig. 4F), and since we also did not find a significant effect of prepulse, we averaged the prepulses together for each mouse and perform a two-way ANOVA on treatment x lighting. We found that poly IC exposure led to significantly decreased percent PPI compared to saline-exposed females using both lots of poly IC (main effect of treatment: lot 1: F_(1, 12)_ = 6.082, *p* = 0.0061; lot 2: F_(1, 14)_ = 1.358, *p* = 0.0485) (Fig. 4E, Supplementary Fig. 4E).

#### Sex differences

To directly address sex differences, we performed three-way ANOVAs for treatment × lighting × sex (Supplementary Table 5).

In the open field test, a significant three-way interaction was found when assessing thigmotaxis (F_(2, 112)_ = 3.699, *p* = 0.0217), where females had significantly higher thigmotaxis than males using both lots (main effect of sex: lot 1: F_(1, 56)_ = 15.37, *p* = 0.0002; lot 2: F_(1, 489)_ = 19.64, *p* = 0.0001). No sex differences were observed in horizontal activity or total distance traveled. In the EPM test, a significant treatment x sex interaction was found in time in closed arms (F_(1, 53)_ = 4.334, *p* = 0.0422), and females spent significantly more time in the closed arms (main effect of sex: F_(1, 53)_ = 22.89, *p* = 0.0001), less percent time in the open arms (main effect of sex: F_(1, 53)_ = 15.31, *p* = 0.0003) and less percent entries in the open arms (main effect of sex: F_(1, 55)_ = 17.44, *p* = 0.0001) compared to males using lot 1 but not lot 2. Finally, in PPI, no sex differences were observed, except females exhibited lower baseline startle response scores than males using lot 1 (main effect of sex: F_(1, 50)_ = 12.41, *p* = 0.0009) and lot 2 (main effect of sex: F_(1, 48)_ = 14.88, *p* = 0.0003).

### Increased microglial morphology values and density by poly IC exposure were attenuated by LL exposure in the dentate gyrus

**Figure 5:**
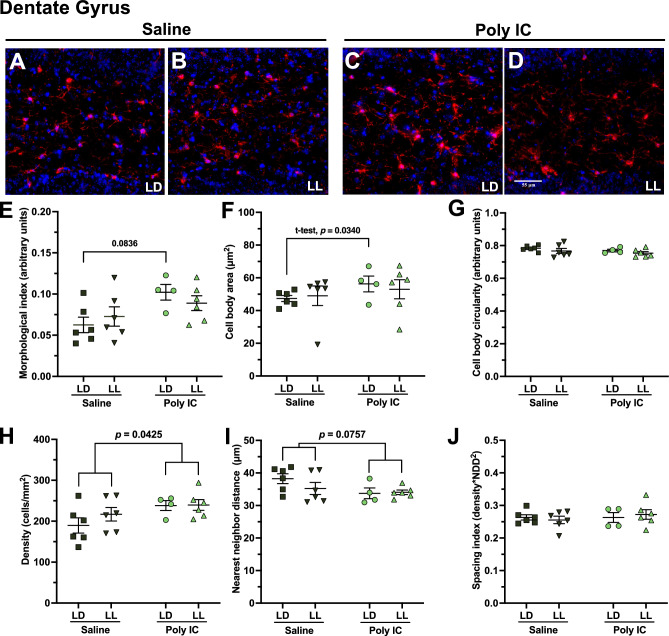
In the dentate gyrus, LL exposure diminished effects on microglia caused by prenatal poly IC. Representative images of microglia from the dentate gyrus for each group under each lighting condition are shown (**A**–**D**). Morphological index (**E**), cell body area (**F**) and cell body circularity (**G**), microglial density (**H**), nearest neighbor distance (**I**), and spacing index (**J**) were assessed in males. Data points represent individual mice and are presented as mean ± SEM. Two-way ANOVAs (factors treatment × lighting with Tukey’s post-hoc comparisons) were conducted.

Representative images of microglia from the DG for each group under each lighting condition are shown (Fig. 5A–D). As reported previously^13^, we confirmed that poly IC exposure had long term effects on microglia in resulting adult offspring. This is exhibited as a significantly increased morphological index in the poly IC-exposed mice compared to saline-exposed mice (main effect of treatment: F_(1, 18)_ = 5.727, *p* = 0.0278), with no significant treatment × lighting interaction (Fig. 5E). Using post hoc tests, we found that under LD, poly IC induced a trending increase in morphological index (saline/LD versus poly IC/LD: *p* = 0.0832), which was not observed after LL exposure (saline/LL versus poly IC/LL) (Fig. 5E). When assessing cell body area, the post hoc test between saline/LD versus poly IC/LD was not significant, but due to a similar pattern being observed as for the morphological index, a t-test was performed and revealed a significant difference (t-test between saline/LD versus poly IC/LD, *p* = 0.0340) (Fig. 5F). No significant differences were observed in cell body circularity (Fig. 5G). Overall, we observed a diminished response of poly IC exposure on adult microglial morphology after LL exposure, compared to LD, and no significant differences in cell body area or cell body circularity.

We next explored microglial density. Density was significantly increased due to poly IC exposure (main effect of treatment: F_(1, 18)_ = 4.766, *p* = 0.0425), with no significant treatment × lighting interaction and no main effect of LL (Fig. 5H). A similar trend was observed when we conducted the nearest neighbor distance (NND) algorithm, which calculates the average distance of each cell to its nearest neighboring cell. Specifically, poly IC exposure led to a trending decrease in NND (main effect of treatment: F_(1, 18)_ = 3.552, *p* = 0.0757), with no treatment × lighting interaction and no main effect of LL (Fig. 5I). Lastly, we saw no significant differences in spacing index, which describes how the cells are distributed in the region of interest (Fig. 5J). Poly IC overall led to an increased density and perhaps a decreased NND in the DG of males.

Representative images of microglia from CA1 and PFC for each treatment under each lighting condition are shown (Supplementary Fig. 5A–D; Supplementary Fig. 6A–D). No differences were observed in the morphological index, cell body area, cell body circularity, density, NND or spacing index (Supplementary Fig. 5E–J; Supplementary Fig. 6E–J).

## Discussion

Our study showed a significant interaction between MIA and environmental circadian disruption, both at the behavioral and cellular levels. In particular, we found that MIA male offspring exhibited decreased sociability specifically after LL. Given that reduced sociability is a common symptom of SCZ^54^ and ASD^55^, this finding aligned with our hypothesis that adulthood LL exposure interacts with pre-existing risk factors for NDDs, such as prenatal infection, to exacerbate behaviors related to NDDs. We also found that poly IC exposure alone led to various group differences, including deficits in PPI across both sexes, which is consistent with the literature on MIA^56^, and is exhibited by individuals with NDDs^52,53^. Females had a much milder phenotype than males, which is also consistent with literature on MIA in rodents^44^. With respect to microglia, we found that poly IC exposure led to increased microglial morphology index and density in the DG, which would indicate a more reactive phenotype, while LL exposure seemed to attenuate these effects.

The behavioral effects observed seem dependent on the lot of poly IC used. Despite using the same dose of poly IC, the cytokine response in maternal serum between poly IC lots differed^33^, which may have influenced the severity of the behavioral deficits in adult offspring. This is unsurprising given that the effects of poly IC are dose-dependent^57,58^ and, in humans, there is a positive correlation between the severity of maternal inflammation and NDDs outcomes^59^. Additionally, the poly IC lot that triggered the stronger cytokine response in dams (lot 1) also led to significant weight loss in adult poly IC-exposed male and female offspring compared to their respective controls.

Microglia are immune cells in the central nervous system, contribute to its development, and rapidly respond to homeostasis disruptions and immune challenges^16^. Microglia continually survey their microenvironment by extending and contracting processes into nearby synapses and are responsible for sculpting synapses during development through processes such as synaptic pruning^60–62^. Studies have visualized and quantified characteristics of microglial activation using PET neuroimaging in vivo and by analyzing post-mortem brain tissue in ASD^63^ and SCZ^64^. Despite mixed results, some patients exhibited characteristics of microglial activation in morphological state and increased density in cortical regions (e.g., PFC, visual cortex), hippocampus and cerebellum^65–69^. In animal studies, poly IC-exposed offspring exhibited increased microglial clustering, reduced arborization and increased “dark” microglia, indicative of a pro-inflammatory state^13^. It is worth noting that “dark” microglia are thought to be a sub-class of microglia that exhibit signs of increased oxidative stress, giving them a dark appearance, and are reported to be abundant under chronic stress^70^. Microglia have circadian clocks; they display 24-h mRNA rhythms of several inflammatory factors and circadian clock genes^71^. Additionally, exposure to abnormal lighting conditions altered microglia cytokine expression following an immune challenge^72,73^ and mice lacking the circadian clock protein REV-ERBα showed activated hippocampal microglia and increased proinflammatory gene transcription^73^. Given these results, and that microglia colonize the fetal brain around E8-9 (i.e., when we injected the poly IC)^14^ and have the capacity to become and remain chronically “primed”^74^, we expected that environmental circadian disruption would heighten the activation-related characteristics of MIA-primed microglia. Surprisingly, we observed the reverse in our microglia data. However, it is possible that protective mechanisms are controlling and mitigating the response of microglia, rendering them tolerant to LL exposure. Future studies would benefit from exploring beyond morphology and density and assess whether microglial functions are similarly affected^75^. Additionally, studying the state and function of microglia across 24 h would also be informative, as well as whether these effects are observed in females.

Protocols of environmental circadian disruption vary between studies. Given that artificial and irregular light schedules are common, we chose to study the effects of LL exposure, meant to mimic light at night. The treatment × lighting interaction that we observed in social behavior may result from a direct effect on the suprachiasmatic nucleus (SCN), a brain region that plays a key role in coordinating circadian oscillations. Namely, LL desynchronizes rhythms of cells of the SCN^76^, and this long term desynchrony may disrupt SCN-driven rhythms in behavior and physiology^77^. Alternatively, LL may exert its effects on behavior by targeting brain regions other than the SCN. For example, light information is primarily transmitted to the brain via intrinsically photosensitive retinal ganglion cells (ipRGCs) in the retina^78,79^. Although ipRGCs innervate the SCN, they also innervate various cortical and limbic areas, including the amygdala^80^, which play a role in social behavior^81^. Lastly, it is possible that LL exerts its effects indirectly by targeting peripheral tissues. Exposure to light at night has many peripheral effects^82^, such as on metabolism^83^, and is known to affect peripheral clock gene expression and circadian hormones^84,85^. Since LL does not affect corticosterone secretion levels^35^, the behavioral effects induced by LL are not likely due to stress. In summary, it is unclear if LL exerts its effects on behavior through circuits involving the SCN, circuits in other brain areas directly impinged upon by ipRGCs, or peripheral effects. An additional consideration is the timing of the circadian disruption; perhaps an early exposure to environmental circadian disruption, such as during adolescence, which is a critical time for brain development, would lead to more pronounced differences.

The multifactorial aspect of NDDs, such as SCZ and ASD, prompts the study of the interaction between multiple risk factors, instead of studying risk factors in isolation. Here, we found interactions between MIA and circadian disruption at the behavioral and microglial level. Our data supports a role for circadian disruption as a risk factor for NDDs, which has already been shown in a genetic mouse model for schizophrenia^35,36^. Future research should address the mechanisms that underlie this interaction to inform the development of circadian-based therapies that aim to prevent or mitigate these serious diseases.

## Supplementary Information

Supplementary Information.

## Data Availability

All processed data are available in the figures of the manuscript. All raw data that support the findings of this study are available from the corresponding authors upon request (e.g., raw data scores from behavioral tests, and .vsi images for microglia data).
