# Supplementary material for: Behavioral and cellular responses to circadian disruption and prenatal immune activation in mice
Source: Sci Rep. 2023 May 13;13:7791. doi: 10.1038/s41598-023-34363-w (PMC10182998; doi:10.1038/s41598-023-34363-w)
Supplement: Supplementary file 1 — Supplementary Information. [file 41598_2023_34363_MOESM1_ESM.pdf]

Behavioral and cellular responses to circadian disruption and prenatal immune activation in mice

Tara C. Delorme, William Ozell-Landry, Nicolas Cermakian, and Lalit K. Srivastava

Supplementary Information

Supplementary Methods

Animals and housing

Male and female C57BL/6J mice for the maternal immune activation (MIA) mating protocol were ordered from Jackson Laboratory (product number: 000664) at 8 weeks old. Male C57BL/6 mice used as strangers for the three-chamber social interaction test were ordered from Charles River Laboratories (Saint-Constant, QC, Canada) and were age and sex matched to the test mice. Offspring resulting from the MIA protocol and controls were placed in light-proof ventilated cabinets (Actimetrics, Wilmette, IL, USA). Cabinet light was controlled via an external timer, with a light intensity of 150-200 lux (cool white LED lighting).

Timed mating protocol for MIA

Male and female mice were placed under standard laboratory lighting (LD; 12 h of light, 12 h of dark) for 2 weeks to acclimate to their surroundings. Mice were mated overnight, and the presence of a vaginal plug was assessed in females within the first hour following lights on. The day of vaginal plug was considered embryonic day 0.5 (E0.5).

Litter details

In the first cohort, 20 female breeders were mated with 10 male breeders: 6 out of 12 poly IC-injected dams gave birth (average litter size: 6.7, total male offspring used = 24, total female offspring used = 8) and 6 out of 8 saline injected dams gave birth (average litter size: 7.8, total male offspring used = 20, total female offspring used = 8). For the second cohort, 19 female breeders were mated with 9 male breeders: 6 out of 11 poly IC-injected dams gave birth (average

litter size: 6.8, total male offspring used = 24, total female offspring used = 8) and 4 out of 8 saline-injected dams gave birth (average litter size: 6.5, total male offspring used = 13, total female offspring used = 8).

Experimental timeline and details

At 3 weeks old, poly IC- and saline-exposed male and female pups were weaned, tagged, and weighed. Two to three sex-matched littermates were group housed, offspring were aged to adulthood (8 weeks old) and were weighed before being placed in light-proof ventilated cabinets, where they were left to acclimatize to their environment for 2 weeks before behavioral tests began. All tests were performed under dim light conditions, where the lighting intensity at the center of the testing chambers was 10 lux. To minimize the effect of litter, mice from 4-6 litters were used in each experimental condition. Additionally, we visually confirmed that the significant effects that we reported were not driven by a cluster of mice from the same litter and ensured effects were still present after averaging individual mouse scores by litter.

Behavioral outcomes

The behavioral tests conducted under LD1 and LD2 were performed in the first 3 hours after the lights turned on (between Zeitgeber Time (ZT) 1-3). When we conducted the behavioral tests under LL, mice were “free running”, meaning they were not entrained to an external stimulus (lights on/lights off). Thus, we used passive infrared sensors that measure general home cage locomotion, to assess when the mice were active versus inactive. The time the mice became active was considered Circadian Time (CT) 12, and CT 0 was defined as half a period (~12 hours) later, calculated using a chi-square periodogram (ClockLab software, version 6,

Actimetrics, Wilmette, IL, USA). Thus, behavioral tests under LL were conducted between CT 1-3 for each cage of mice. Behavioral tests were performed in order of least stressful to the most (i.e., open field followed by EPM, three-chamber social interaction, and PPI). Two rest days were scheduled between each test. In the EPM and three-chamber social interaction test, we used Swann cameras (DVR4-4575, 4 Channel digital video recorder & 2x PRO-T853 Cameras) to record behavior and the TopScan 2.0 software (Clever Sys, Restin, VA, USA) to track mouse movement.

Open field test

We used the VersaMax Legacy Open Field setup (AccuScan Instruments, Inc., Columbus, OH, USA) system, which consisted of acrylic chambers (Length × Width × Height = 17.5 cm × 10 cm x 26 cm) equipped with infrared sensors to record and score locomotion-related variables. Mice were free to explore the chamber for 50 minutes. Data were collected using the Versamax Software (version 4.0, 2004; AccuScan Instruments, Inc.).

Elevated plus maze (EPM) test

The apparatus was elevated 70 cm above the ground. The center area measured 5 cm × 5 cm, and each of the 4 arms measured Length = 50 cm × Width = 5 cm. Mice were placed in the intersection of the four arms, facing an open arm. Mice could freely explore the chamber for 5 minutes after which they were returned to their home cage. EPM is based on the conflict between the rodent’s preference for enclosed areas (the closed arms) and their innate motivation to explore a novel environment (the open arms). A video camera positioned directly above the apparatus recorded the session, and time spent and entries in each arm was scored. Mice that jumped off the

maze were excluded from the analysis. Higher scores on the EPM formula indicated less anxiety-like behavior. Two male saline-exposed mice from lot 1 and 1 male saline-exposed mouse from lot 2 jumped off the maze and were excluded from the analysis.

Three-chamber social interaction test

The test is comprised of 3 consecutive phases, each lasting 10 minutes. In the *habituation phase*, mice could freely explore the testing apparatus, which consisted of a three-chamber plastic apparatus (Length = 26 cm x Width = 21.6 x Height = 21.6 cm) with an empty wire container (Diameter = 7.6 cm, Height = 9.5 cm) in each of the two extreme chambers. The habituation formula is shown below. A value of 0.5 indicates no preference for either chamber. A value between 0.5-1 indicates a preference for Zone 1 and a value between 0-0.5 indicates a preference for Zone 2.

$$Habituation\ formula = \frac{\text{Time spent in Zone 1}}{\text{Time spent in Zone 1+Time spent in Zone 2}}$$

In the *social preference formula*, a value of 0.5 indicates no preference for either chamber. A value between 0.5-1 indicates a preference for stranger 1 (i.e., social preference) and a value between 0-0.5 indicates a preference for the object. The object used was a similar size to the stranger mice. Mice typically prefer to explore a mouse over an object.

In the *social memory formula*, a value of 0.5 indicates no preference for either chamber. A value between 0.5-1 indicates a preference for stranger 2 (i.e., social memory) and a value between 0-0.5 indicates a preference for the familiar stranger 1. Mice typically prefer to explore a novel mouse over a familiar one.

Stranger mice

The stranger mice were strain-, sex-, and aged-matched to the test mice and were habituated to the chamber and wire containers for 30 minutes the day before the test. The location of the stranger mice and the object was counterbalanced. A video camera positioned directly above the apparatus recorded the 3 phases, and time in each chamber was scored.

Prepulse inhibition of acoustic startle (PPI)

PPI was measured using commercially available startle chambers (San Diego Instruments, San Diego, CA, USA), and a commercial software package by SR-LAB. Mice were placed into a cylindrical Plexiglass enclosure (Diameter = 8 cm, Length = 16 cm), mounted on a Plexiglass base, within a dimly lit (10 lux) and sound-attenuating chamber. The speaker that delivered the acoustic stimuli was in the ceiling of the chamber (24 cm above the animal) and delivered a 70-dB background noise. A piezoelectric accelerometer fixed to the Plexiglass base was used to detect and transduce motion resulting from the animal's startle response. The session began with a 5-minute acclimation period. Each session consisted of 50 trials. In the first 6 and final 6 trials, a 'startle noise burst' (120-dB for 50ms, broad-band burst) was played in the absence of a prepulse (called 'startle-only' trials). The "baseline startle response" was calculated by averaging the startle magnitude after the last 'startle-only' trials. For the middle 38 trials, 8 trials were 'startle-only' trials, 5 trials had no stimulus, and 25 trials had the startle noise burst preceded by a prepulse stimulus (30 ms, broad-band burst) that was either 6, 9, 12 or 15 dB above the background noise and presented 100 ms before the startle pulse. The order of the middle 38 trials varied randomly. The intertrial interval also varied randomly, with an average intertrial interval of 15 seconds. The higher the percent PPI, the greater the inhibition to the startle when preceded by a prepulse. In lot

1, 3 male saline-exposed and 4 male poly IC-exposed mice were excluded and in lot 2, 1 male saline-exposed mouse was excluded due to issues with the recording apparatus.

Microglia characterization

Tissue preparation

Mice were anesthetized with an intraperitoneal injection of ketamine/xylazine/acepromazine between ZT 1 and 3. Mice were transcardially perfused with ice-cold phosphate buffered saline (PBS), followed by 4% paraformaldehyde (# 31985-062, Invitrogen). Brains were post-fixed for 24 hours in 4% paraformaldehyde at 4°C, then transferred to a 30% sucrose solution for 48 hours at 4°C. Brains were placed in OCT compound and flash frozen in isopentane over dry ice before being stored at -80°C. Brains were sliced with a cryostat at 30 μm section. Sagittal sections containing Bregma 0.36 to 1.00 (which included the dorsal hippocampus and the medial prefrontal cortex), based on the stereotaxic atlas of Paxinos and Franklin (4th edition), were processed for immunofluorescence staining and imaging.

Immunohistochemistry

Slices were washed in PBS and Triton 0.2% and blocked in donkey serum (1:10, including 0.2% Triton X-100 and PBS) at room temperature for 1 hour to prevent non-specific antibody binding. Slices were incubated with primary antibody [1:1000, Rabbit anti-Iba1 (ionized calcium binding adaptor molecule 1, a marker for microglia), # 019-19741, Cedarlane] overnight at 4°C. Slices were rinsed in PBS and Tween 0.1% before they were incubated in secondary antibody (1:1000, Alexa fluor 594 Donkey anti-rabbit IgG, # 711-585-152, Cedarlane) at room temperature for 2 hours. All primary and secondary antibodies were diluted in the blocking

buffer mentioned above. Sections were washed in PBS and Tween 0.1%, counter-stained with DAPI (1:20000, # D9542, Sigma), and mounted with antifade mounting medium (H-1000, lot # T0606, Vector Laboratories) under a glass coverslip.

Microglia visualization and analysis

Brain sections were visualized using an Olympus BX63 automated fluorescence microscope. We characterized the microglia based on morphology, density, and spacing.

Morphology

Measures of morphology included soma area and perimeter, arborization area and perimeter and soma circularity. Arborization area and process perimeter were measured using the 'polygon' tool to trace around the edge of all the processes of a single microglia. To determine soma area, soma perimeter, and soma circularity, the 'freehand' tool was used to trace around the cell body seen on the Iba1 stain. Soma circularity was calculated using the 'shape descriptors' tool. A morphological index was calculated by dividing the soma area by the arborization area for each cell.

Density

To determine the density of microglia per image, we first used the 'freehand' tool to trace a region of interest. The 'threshold' tool was used to identify each microglia, and the 'analyze particles' function was used to automatically record the number of identified microglia, as well as each of their spatial coordinates. The 'nearest neighbor distance' plugin was then applied to determine the distance between each cell and its closest neighbor. The density of microglia for

each image was calculated by dividing the number of cells in that image by the region of interest identified. A spacing index was calculated by multiplying the average density for each animal obtained above by the square of the average nearest neighbor distance.

Statistics

For the behavioral data (**Fig. 2-4, Supplementary Fig. 2-4**), 2-way mixed effect ANOVAs were conducted using Tukey's post hoc comparisons, with treatment (poly IC or saline) as a between-subjects variable and lighting (LD1, LL, and LD2) as a within-subjects variable. Main effects were explored if no significant treatment x lighting interaction was observed. Including lighting as a within-subjects variable allows for the same mice to be tested throughout the experiment, but it does introduce the likelihood that mice habituate to the test, which is a limitation of this design. Three-way ANOVAs were also conducted (using SPSS statistical software version 29) for prepulse inhibition (3rd factor: prepulse) and locomotion (3rd factor: time). For the microglia data (**Fig. 5, Supplementary Fig. 5-6**), 2-way mixed effect ANOVAs were conducted using Tukey's post hoc comparisons, with treatment (poly IC or saline) and lighting condition (LD or LL) both as between-subjects variables. If no interaction was found, main effects were explored. The data describing number of pups per litter (**Supplementary Fig. 1A-B)**, passed the Shapiro-Wilk normality test and had equal variances (assessed by an F-test); thus, independent samples t-test was used between poly IC- and saline-injected pregnant dams. For offspring weight data (**Supplementary Fig. 1C-F)**, we conducted 2-way mixed effect ANOVAs, with treatment (poly IC or saline) as a between-subjects variable and age (weaning and adulthood) as a within-subjects variable. We explored treatment x age interactions and performed Tukey's post hoc comparisons when applicable. The data describing sex differences

(**Supplementary Table 5)** were assessed using three-way ANOVAs (treatment x lighting x sex) and decomposed by assessing the 2-way interaction (treatment x sex) and observing the main effect of sex. No outliers were identified (ROUT's test, Q = 1%).

Supplementary Figures

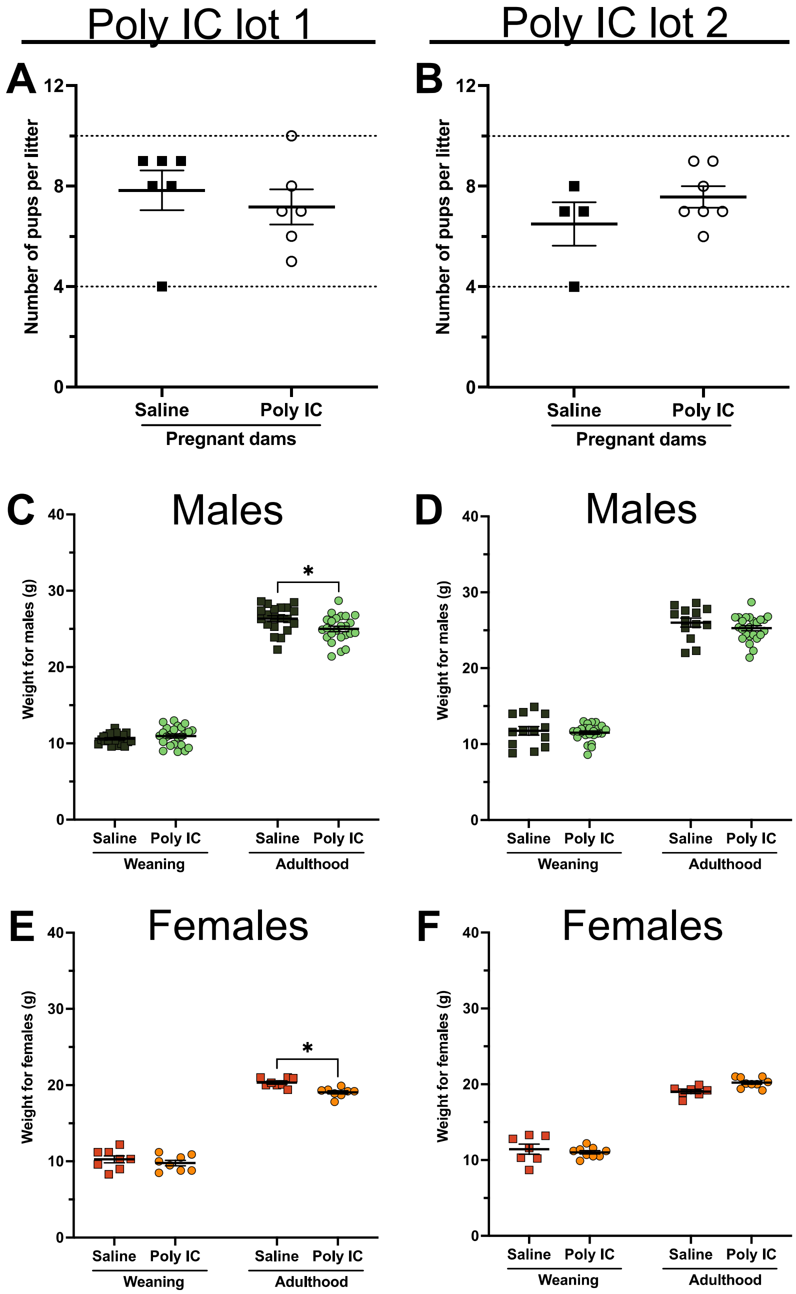

Supplementary Fig. 1. Litter details and poly IC- and saline-exposed offspring body weights at weaning and adulthood. Number of pups from each poly IC and saline litter (**A**). Litters had a minimum of 4 pups/litters and a maximum of 10 pups/litter. Individual points represent a litter and data are represented as mean ± SEM. Independent samples t-tests were conducted. Body weight between groups at weaning (3 weeks old) (**B**) and adulthood (8 weeks old) (**C**) are presented. Data points represent independent offspring and data are represented as mean ± SEM. Two-way ANOVAs (factors treatment x lighting with Tukey's post hoc comparisons) were conducted. *$p < 0.05$ (post hoc).

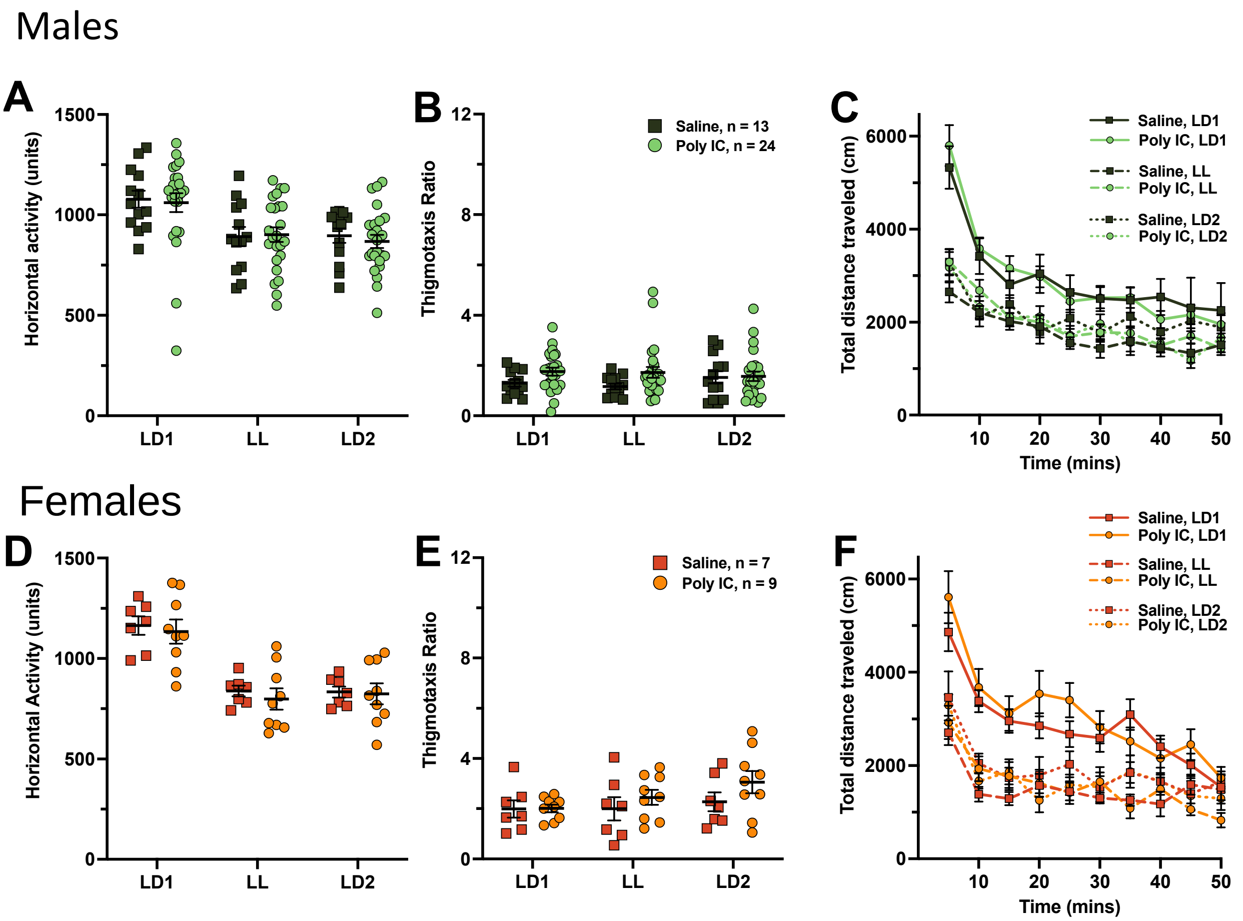

Supplementary Fig. 2. No significant effects in spontaneous locomotion while using poly IC lot 2. Spontaneous locomotor activity was measured in the open field test. Horizontal activity **(A, D)**, thigmotaxis **(B, E)** and total distance traveled (**C, F**) were assessed in males (**A-C**) and females (**D-F**). For panels **A, B, D and E** data points represent individual mice, and are presented as mean ± SEM. Two-way ANOVAs (factors treatment x lighting with Tukey's post hoc comparisons) were conducted. For panels **C** and **F,** group averages ± SEM are shown over each 10-minute bin of the test. Three-way ANOVAs (factors treatment x lighting x time) were conducted. See **Supplementary Table 3 and 4** for full statistics.

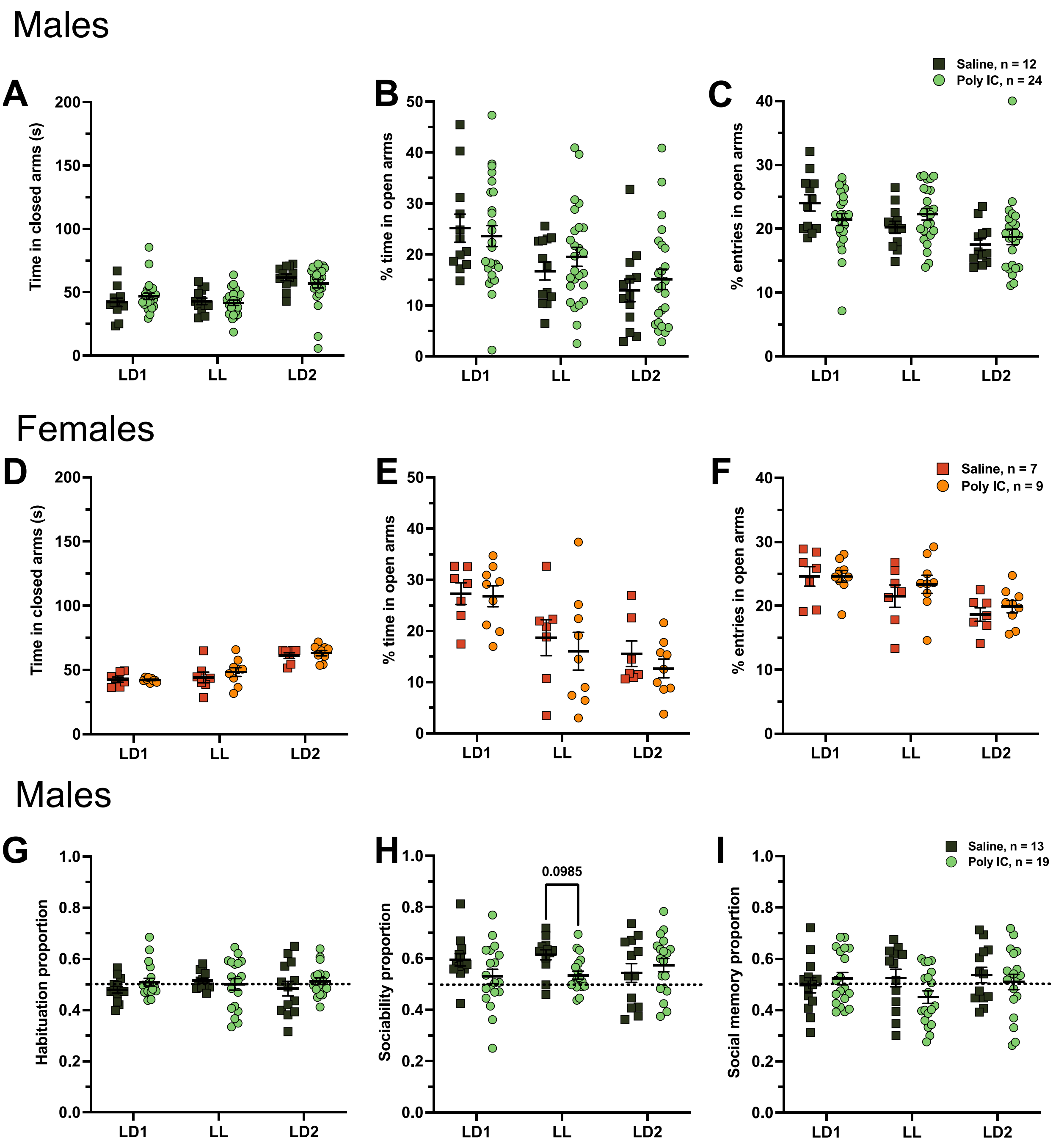

Supplementary Fig. 3. No differences in anxiety-like behavior, and a trending effect for poly IC-exposed males to exhibit reduced sociability after LL. In the elevated plus maze, time in closed arms (**A, D**), percent time in open arms (**B, E**) and percent entries in open arms (**C, F**) were assessed in males (**A-C**) and females (**D-F**). In the three-chamber social interaction test, preference proportions were assessed for the habituation phase **(G)**, sociability phase (**H**) and social memory phase (**I**) in males. Data points represent individual mice, and are presented as mean ± SEM. Two-way ANOVAs (factors treatment x lighting with Tukey's post hoc comparisons) were conducted. See **Supplementary Table 3 and 4** for full statistics.

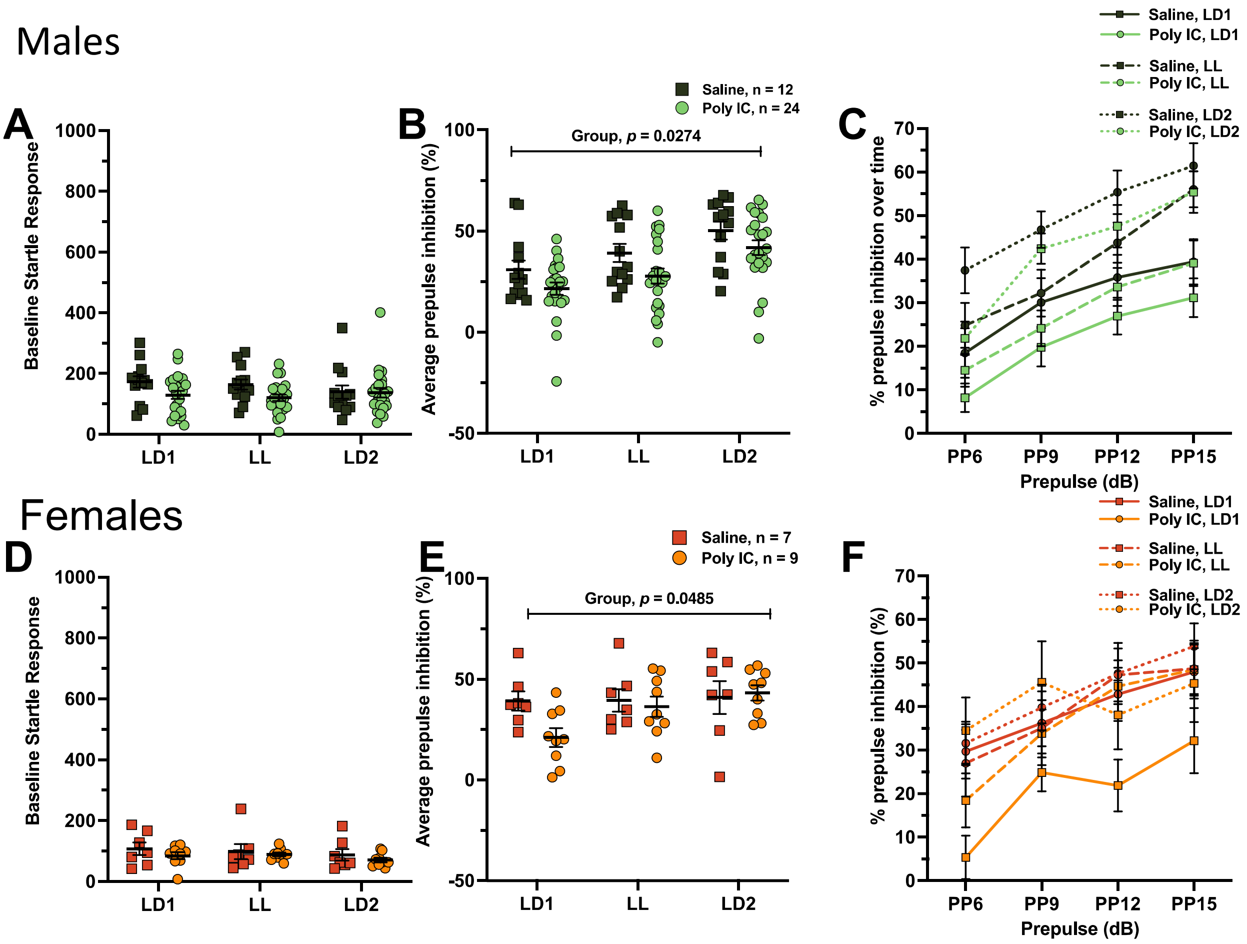

Supplementary Fig. 4. Deficits in PPI following prenatal poly IC exposure. Prepulse inhibition of acoustic startle (PPI) was used to assess sensory motor gating. Baseline startle response (**A, D)**, average PPI (%) (**B, E**) and PPI (%) across each prepulse levels (**C, F**) were assessed in males (**A-C**) and females (**D-F**). For panels **A, B, D and E** data points represent individual mice, and are presented as mean ± SEM. Two-way ANOVAs (factors treatment x lighting with Tukey's post hoc comparisons) were conducted. For panels **C** and **F,** group averages ± SEM are shown over prepulse level. Three-way ANOVAs (factors treatment x lighting x time) were conducted. See **Supplementary Table 3 and 4** for full statistics.

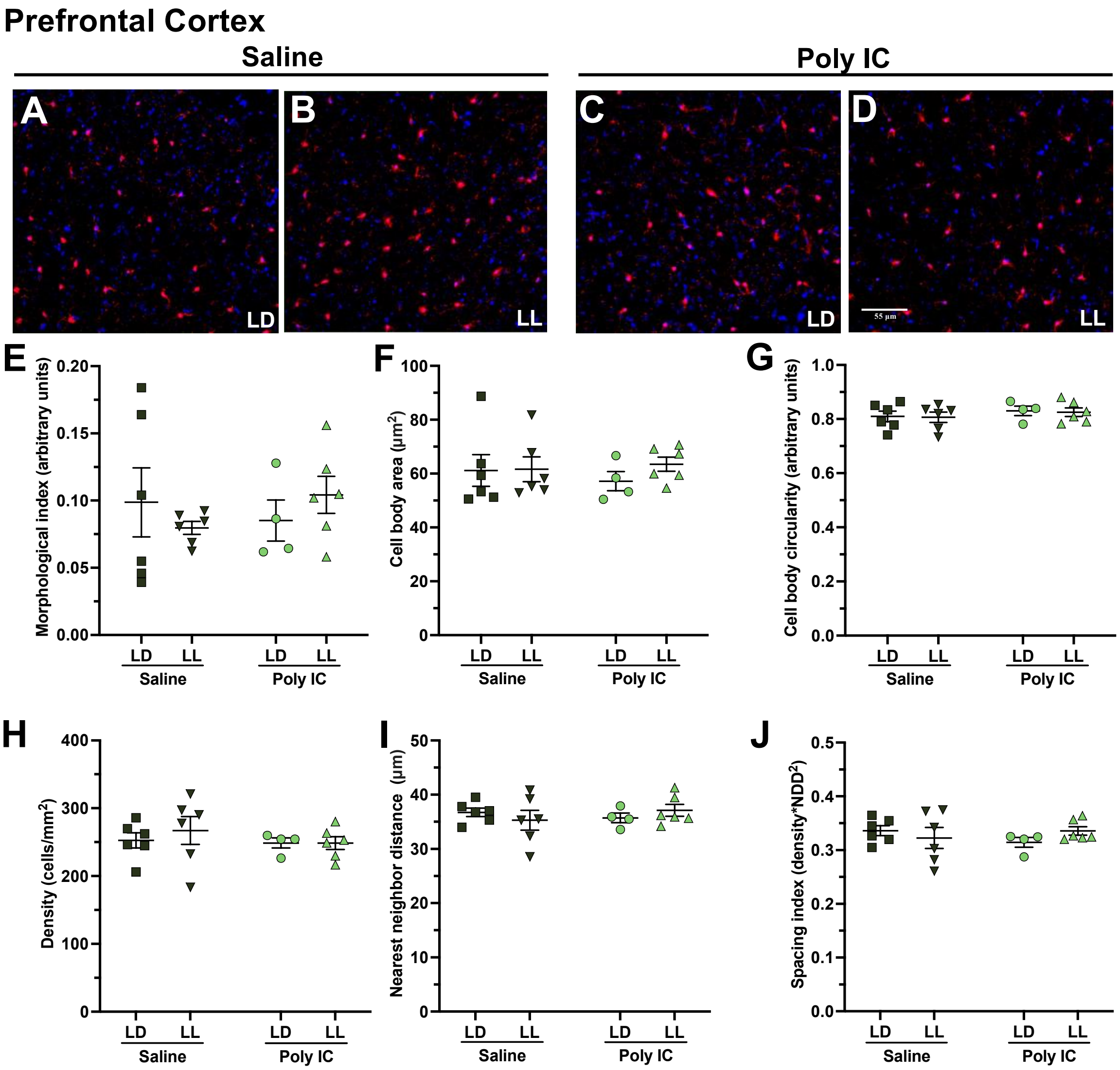

Supplementary Fig. 5. In the PFC, no morphological or density-related differences in microglia were observed. Representative images of microglia from the PFC for each group under each lighting condition are shown **(A-D)**. Morphological index (**E**), cell body area (**F**) and cell body circularity (**G**), microglial density (**H**), nearest neighbor distance (**I**), and spacing index (**J**) were assessed in males. Data points represent individual mice, and are presented as mean ± SEM. Two-way ANOVAs (factors treatment x lighting with Tukey's post hoc comparisons) were conducted.

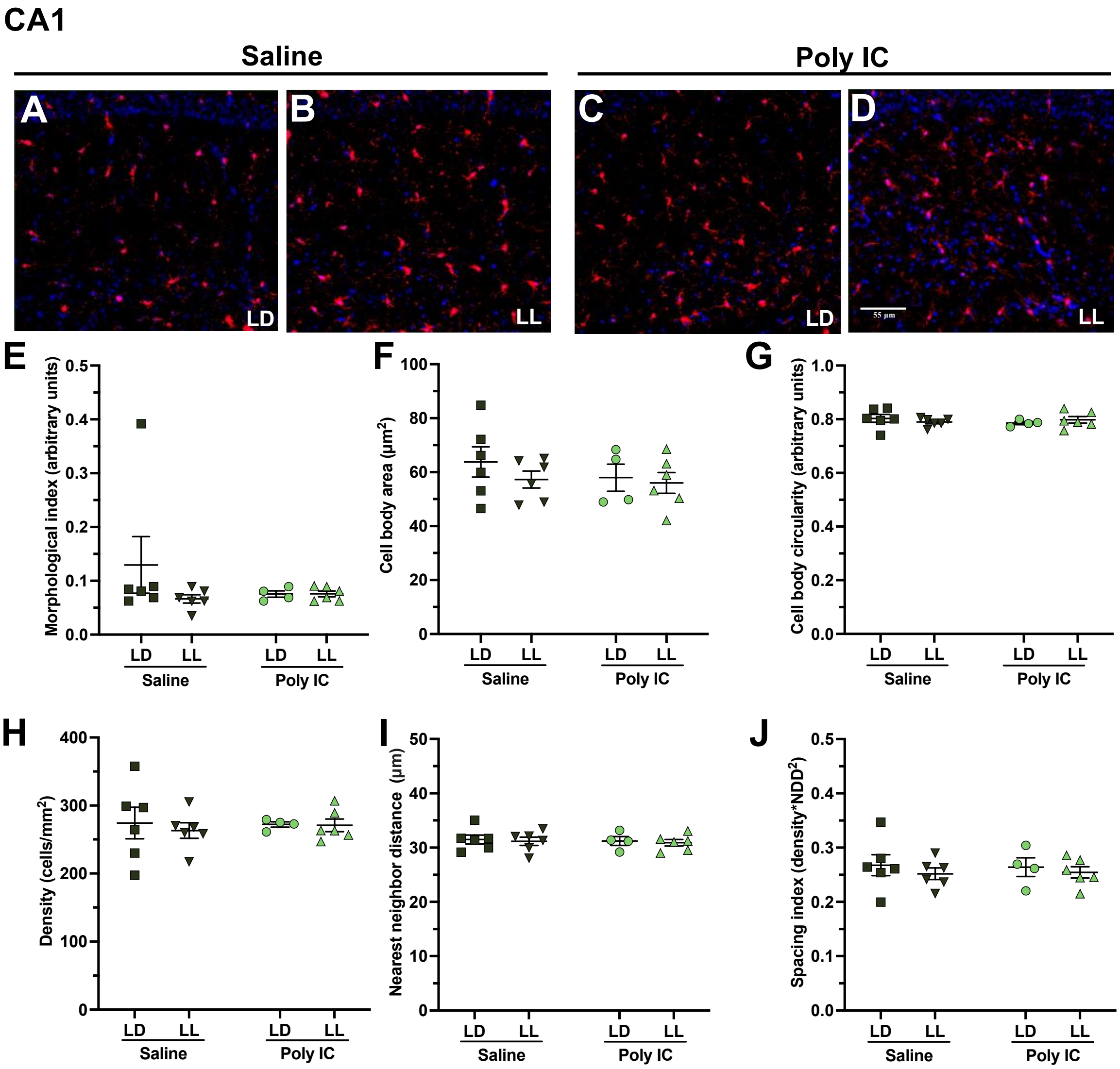

Supplementary Fig. 6. In area CA1, no differences were observed in microglia after poly IC alone, LL alone or the combined effects of both poly IC and LL. Representative images of microglia from the CA1 for each treatment under each lighting condition are shown **(A-D)**. Morphological index (**E**), cell body area (**F**) and cell body circularity (**G**), microglial density (**H**), nearest neighbor distance (**I**), and spacing index (**J**) were assessed in males. Data points represent individual mice, and are presented as mean ± SEM. Two-way ANOVAs (factors treatment x lighting with Tukey's post hoc comparisons) were conducted.

Supplementary Table 1. Poly IC and saline-exposed males (cohort 1).

	Treatment x lighting		Treatment		Multiple comparisons *p*-values		
	F	*p* value	F	*p* value	LD1	LL	LD2
Open field							
Horizontal activity	1.121	0.3308	6.233	**0.0165**	**0.0456**	0.9999	0.1055
Thigmotaxis ratio	0.5049	0.6054	0.6147	0.4374			
Total distance traveled	0.3186	0.7281	7.398	**0.0095**	*0.0741*	0.5920	0.1417
Elevated plus maze							
Time in closed arms	0.2992	0.7422	0.7579	0.3892			
Percent in open arms	0.2075	0.8131	1.241	0.2719			
Entries in open arms	1.081	0.3441	1.013	0.3201			
Three-chamber social interaction							
Habituation ratio	1.284	0.2818	0.0760	0.7834			
Sociability ratio	2.575	***0.0846***	5.365	**0.0276**	0.9999	**0.0064**	0.9999
Social memory ratio	0.4207	0.6579	0.4752	0.4924			
Prepulse inhibition of acoustic startle							
Baseline startle response	0.2655	0.7689	1.889	0.1925			
Null response	1.228	0.3094	3.414	0.0875			
Average PPI	0.5246	0.5979	8.158	**0.0135**	0.2015	0.1891	0.6034

Notes: Results from two-way ANOVAs (treatment x lighting) for behavioral parameters from poly IC and saline-exposed males (cohort 1). ANOVA factors include treatment (poly IC, saline) and lighting (LD1, LL, LD2). P-values in **bold** represent significance, and p-values in ***bold and italics*** represent trending significance.

Supplementary Table 2. Poly IC and saline-exposed females (cohort 1).

	Treatment x lighting		Treatment		Multiple comparisons *p*-values		
	F	*p* value	F	*p* value	LD1	LL	LD2
Open field							
Horizontal activity	0.2626	0.7710	1.070	0.3185			
Thigmotaxis ratio	1.957	0.1602	0.6185	0.4447			
Total distance traveled	0.3498	0.7078	0.0761	0.7886			
Elevated plus maze							
Time in closed arms	0.0336	0.9670	14.63	**0.0019**	0.1709	0.3618	0.1988
Percent time open arms	1.083	0.3523	2.729	0.1208			
Percent entries open arms	0.1307	0.8778	1.188	0.2820			
Prepulse inhibition of acoustic startle							
Baseline startle response	0.1514	0.8602	2.463	0.1389			
Null response	1.228	0.3094	3.414	0.0875			
Average PPI	0.0445	0.9565	10.40	**0.0061**	0.1107	0.2148	0.3625

Notes: Results from two-way ANOVAs (treatment x lighting) for behavioral parameters from poly IC and saline-exposed females (cohort 1). ANOVA factors include treatment (poly IC, saline) and lighting (LD1, LL, LD2). P-values in **bold** represent significance.

Supplementary Table 3. Poly IC and saline-exposed males (cohort 2).

	Treatment x lighting		Treatment		Multiple comparisons *p*-values		
	F	*p* value	F	*p* value	LD1	LL	LD2
Open field							
Horizontal activity	0.1392	0.8703	0.0721	0.7899			
Thigmotaxis ratio	1.225	0.2999	2.944	0.0951			
Total distance traveled	0.4777	0.6222	0.0009	0.9754			
Elevated plus maze							
Time in closed arms	1.711	0.1882	0.0011	0.9742			
Percent in open arms	0.5300	0.5909	0.3528	0.5561			
Entries in open arms	2.602	***0.0813***	0.0067	0.9352	0.9999	0.9999	0.2475
Three-chamber social interaction							
Habituation ratio	0.8884	0.4167	0.9791	0.3303			
Sociability ratio	2.903	*0.0626*	2.665	0.1130	0.2553	*0.0985*	0.9999
Social memory ratio	2.814	0.0679	0.5923	0.4475			
Prepulse inhibition of acoustic startle							
Baseline startle response	1.670	0.1959	2.882	0.0987			
Null response	0.2325	0.7932	1.921	0.1748			
Average PPI	0.9913	0.9058	5.314	**0.0274**			

Notes: Results from two-way ANOVAs (treatment x lighting) for behavioral parameters from poly IC and saline-exposed males (cohort 2). ANOVA factors include treatment (poly IC, saline) and lighting (LD1, LL, LD2). P-values in **bold** represent significance, and p-values in ***bold and italics*** represent trending significance.

Supplementary Table 4. Poly IC and saline-exposed females (cohort 2).

	Treatment x lighting		Treatment		Multiple comparisons *p*-values		
	F	*p* value	F	*p* value	LD1	LL	LD2
Open field							
Horizontal activity	0.1265	0.8817	0.2458	0.6277			
Thigmotaxis ratio	0.4366	0.6505	1.473	0.2450			
Total distance traveled	3.161	***0.0579***	0.9729	3.407	***0.0983***	0.9999	0.9999
Elevated plus maze							
Time in closed arms	0.2639	0.7700	0.7849	0.3888			
Percent in open arms	0.1771	0.8386	0.8595	0.3677			
Entries in open arms	0.5131	0.6043	0.4869	0.4953			
Prepulse inhibition of acoustic startle							
Baseline startle response	0.1876	0.8299	0.9038	0.3579			
Null response	0.3736	0.6916	2.316	0.1503			
Percent PPI	2.657	***0.0878***	1.358	0.2633	***0.0642***	0.9999	0.9999

Notes: Results from two-way ANOVAs (treatment x lighting) for behavioral parameters from poly IC and saline-exposed females (cohort 2). ANOVA factors include treatment (poly IC, saline) and lighting (LD1, LL, LD2). P-values in ***bold and italics*** represent trending significance.

Supplementary Table 5. Sex differences statistics from poly IC and saline-exposed mice.

	Cohort 1						Cohort 2					
	Treatment x Sex x Lighting		Treatment x Sex		Main Effect Sex		Treatment x Sex x Lighting		Treatment x Sex		Main Effect of Sex	
	F	*p-value*	F	*p-value*	F	*p-value*	F	*p-value*	F	p-value	F	p-value
Open field												
Horizontal activity	0.9857	0.3764	2.497	0.1197	2.070	0.1558	0.0233	0.9770	0.0182	0.8932	0.2762	0.6016
Thigmotaxis ratio	3.966	**0.0217**	0.9519	0.3334	15.37	**0.0002**	0.6026	0.5494	3.987	***0.0514***	19.64	**0.0001**
Total distance traveled	0.3353	0.7158	0.8703	0.3549	0.050	0.8226	0.7899	0.4567	0.4038	0.5281	0.6017	0.4417
Elevated plus maze												
Time in closed arms	0.2433	0.7844	4.334	**0.0422**	22.89	**0.0001**	0.9537	0.3890	0.2298	0.6338	0.3918	0.5343
Percent in open arms	0.4060	0.6673	1.222	0.2739	15.31	**0.0003**	0.4148	0.6617	1.128	0.2934	0.0550	0.8155
Entries in open arms	0.5478	0.5799	2.194	0.1443	17.44	**0.0001**	0.6839	0.3814	0.1210	0.7295	2.627	0.1116
Prepulse inhibition												
Baseline startle response	0.0193	0.9809	0.1920	0.6631	12.41	**0.0009**	0.7600	0.4705	0.2227	0.6391	14.88	**0.0003**
Average PPI	0.0403	0.9605	1.084	0.3024	0.010	0.9207	1.651	0.1972	0.2288	0.6346	0.1630	0.6882

Notes: Results from three-way ANOVAs (treatment x lighting x sex) for behavioral parameters from poly IC and saline-exposed mice, while also highlighting the treatment x sex interactions and main effects of sex analysis. ANOVA factors include sex (male, female), treatment (poly IC, saline exposure) and lighting (LD1, LL, LD2). P-values in **bold** represent significance, and p-values in ***bold and italics*** represent trending significance.
